# Quantitative Evaluation of Articular Involvement of Posterior Malleolus Associated with Operative Indication: A Comparative Study of Six Methods Based on Radiography and CT

**DOI:** 10.1155/2020/6745626

**Published:** 2020-01-02

**Authors:** Kun Zhang, Xiaoyang Jia, Minfei Qiang, Song Chen, Shuguang Wang, Dongdong Wang, Yanxi Chen

**Affiliations:** ^1^Department of Orthopedic Trauma, East Hospital, Tongji University School of Medicine, 150 Jimo Rd, 200120 Shanghai, China; ^2^Department of Orthopaedic Surgery, Zhongshan Hospital, Fudan University, 180 Fenglin Road, Shanghai 200032, China

## Abstract

The aim of this study was to compare the values of six methods in measuring the involvement of posterior malleolus and to demonstrate the reliability and reproducibility of each method. Three independent orthopaedic surgeons, retrospectively, measured 106 cases. The difference between the six methods was analyzed using Bonferroni-corrected paired *t*-tests after one-way ANOVA. The agreement between the six methods was analyzed using Bland–Altman analysis. The intraclass correlation coefficient (ICC) was used to assess intraobserver reproducibility and interobserver reliability. Significant differences were observed between values of any two of the six measurement methods (*P* < 0.0033), except between any two of the plane radiograph linear, axial CT linear, sagittal CT linear, and 3D CT linear. The Bland–Altman plots demonstrated poor agreement between values of any two of the six methods. The lowest intraobserver reproducibility was 0.46 (moderate) for resident surgeon using plain radiographs. The intraobserver reproducibility for three surgeons using two-dimensional (2D) and 3D images was almost perfect (ICC, 0.82–0.96). The lowest interobserver reliability was 0.41 (moderate) between chief and attending surgeon using plain radiographs, and it improved to almost perfect (ICC, 0.81–0.95) with the use of 3D CT images. The standard error of measurement showed almost the same results as ICC values. The existing operative indications which were determined based on plain radiography are neither reliable nor suitable for other measurement methods. Both 3D linear and 3D surface measurement methods are reliable and reproducible in measuring posterior fragment involvement, and experience is not so crucial. Operative indications for posterior malleolar fractures need to be redefined based on the 3D measurement method.

## 1. Introduction

Ankle fractures are among the most common lower limb fractures, accounting for about 9% of all fractures [1]. Posterior malleolar fractures comprise 14–42% of all ankle fractures [2]. Recently, many scholars recommended anatomical reduction and internal fixation for treatment of ankle fractures [3–5]. But still no consensus was achieved on the operative indication for posterior malleolar fractures [6].

Several scholars suggested operative fixation of posterior malleolar fractures when more than 25% of the tibial plafond is involved [7–9]. A biomechanical study also demonstrated that with fractures constituting 25% of the lateral joint line or more, the normal dynamics of the joint were disrupted [10]. In the meanwhile, some scholars offered different operative indications. A biomechanical study involving 16 cadaveric specimens suggested operation when >33% of the joint is involved based on their findings that displaced posterior malleolar fractures produce a significant decrease in contact area with 33% or greater involvement of the joint [11]. Other indications include >30% of the joint is involved with >2 mm displacement after closed reduction of the ankle [12], >20% of the joint is involved [13], >10% of the joint is involved [14], etc. Although the indications were different, they were all based on a specific percentage. Therefore, precise estimate of the articular involvement of posterior malleolar fractures is crucial for orthopaedic surgeons to decide clinical treatment and judge prognosis [9].

Generally, the size of posterior malleolar fragments was assessed using plain radiographs. However, radiographs may be restricted by the orientation of the foot because of pain and swelling in the acute injury. Surgeons frequently failed to identify posterior malleolar fractures and precisely estimate the size of posterior malleolar fragments when interpreting plain radiographs [15–19]. Axial CT was also used to measure the percentage of posterior malleolar fractures [16, 20]. But two-dimensional (2D) CT still has limitations in assessment. The selection of the observation plane was also affected by the position of the ankle and the experience of observers. A study tried to use three-dimensional (3D) CT to assess the articular involvement of posterior malleolar fractures [21]. But the method adopted, which needs to integrate several sets of software, was complex and time-consuming. The method could hardly be applied to measure large sample and be replicated by other scholars. So up to now, 3D CT has not been extensively applied in posterior malleolar fracture evaluation nor has it been reported in a study on large sample [22, 23]. With the upgrading of computer technology, an efficient system for computer-assisted preoperative planning has been developed [24–26]. The use of computer technology enables multilevel and multiangle evaluation of fracture planes. Surgeons can also perform a virtual operation efficiently and conveniently, including reducing the fracture fragments and selecting a suitable internal fixation device [24, 25]. With this technology, detailed evaluation and accurate measurement of posterior malleolar fracture may be promoted to a higher level. If the measurement method is not reliable or reproducible, large sample and multicenter studies cannot be carried out, and the operative indications which were summarized from the unreliable results will have no reference value. Therefore, the comparison of the results, reliability, and reproducibility of various methods for measuring the articular involvement of posterior malleolar fractures is of great significance. To our knowledge, little literature is available comparing different kinds of methods in measuring the articular involvement of posterior malleolar fractures.

The objective of the study was (1) to explore the difference of 6 methods (plain radiograph linear, axial CT linear, sagittal CT linear, axial CT plane, 3D CT linear, and 3D CT surface) in measuring the involvement of Haraguchi type I posterior malleolar fragment based on a computer-assisted preoperative planning system and large sample and (2) to demonstrate the reliability and reproducibility of each method.

## 2. Materials and Methods

### 2.1. Patient Demographic Data

**Figure 1:**
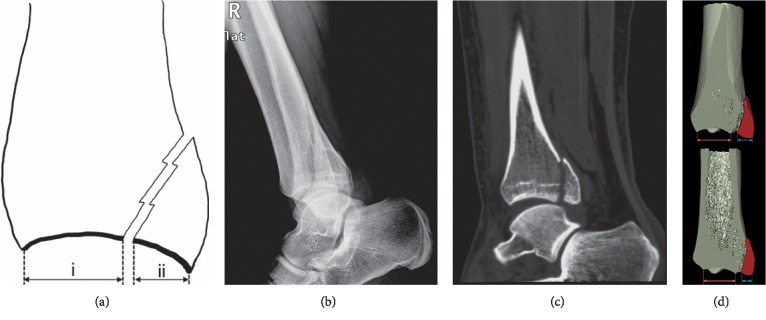
(a) Traditional measurement method on lateral plain radiographs is shown schematically. The percentage of involved articular surface is ii/i + ii. (b) A case would be excluded if the fracture line could not be identified and therefore measurement could not be performed. Images showed the lateral plain radiograph of a 23-year-old male patient with Lauge-Hansen supination-external rotation type 3 ankle fracture. The patient was excluded because the posterior malleolar fracture line was difficult to be identified on the lateral radiograph. (c) On the sagittal multiplanar reconstruction CT image of the same patient, the posterior malleolar fracture line could be clearly identified. (d) A large difference in the articular involvement of posterior malleolar fractures is shown on the sagittal CT images (3D pseudocolor display mode) selected by different observers.

The research project was approved by the Ethics Committee of our hospital (Ethics number 2012–020), and informed consent was obtained. Trauma patients were retrospectively reviewed at our hospital between May 2009 and December 2015. The inclusion criteria were ankle fracture with posterior malleolar fragment, which was confirmed by CT or surgery, between the ages of 20 and 75. The exclusion criteria were patients with pathologic fractures, Haraguchi type II or III posterior malleolar fractures or without standard lateral radiographs, or 16-row spiral CT examinations. Patients were also excluded if posterior malleolar fractures could not be identified on lateral radiographs, or measurement could not be performed on 3D CT images. A total of 235 patients met the inclusion criteria. Fifty-two patients with Haraguchi type II posterior malleolar fractures were excluded. Twenty-five patients were excluded for lack of CT data. Thirty-nine patients were excluded because posterior malleolar fractures could not be identified on lateral radiographs (Figure 1). Twenty-five patients were excluded because measurement could not be performed on 3D CT images. Measurement could not be performed because of severe comminution of articular surface or defect of the posterior malleolar fragments or very small shell-shaped Haraguchi type III fractures. Measurements could not be performed on both lateral radiographs and 3D CT images in 12 patients mentioned above. The remaining 106 patients were finally analyzed (Table 1). All cases were classified based on CT scans according to AO/OTA (Arbeitsgemeinschaft für Osteosynthesefragen/Orthopaedic Trauma Association) and Lauge-Hansen Classification.

### 2.2. Image Evaluation

The X-ray and CT scanning data (DICOM 3.0 format) of all patients were collected. The data of all research subjects were firstly uploaded to picture archiving and communication system (PACS) of the hospital and then imported into the computer-assisted orthopaedic research system (SuperImage orthopaedics edition 1.1, Cybermed Ltd, Shanghai, China) [24].

All the cases were evaluated by 3 independent orthopaedic surgeons (one chief surgeon with 18 years of image reading and clinical experience, one attending surgeon with 9 years of experience, and one resident surgeon with 5 years of experience). The examiners were asked to measure using different methods in 6 phases. There was an interval of 2 weeks between each phase. All observers were blinded to the others' analysis. The measurements were repeated by three observers at an interval of 4 weeks.

**Figure 2:**
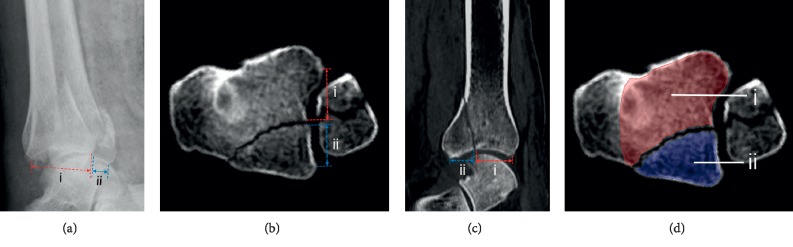
Measurement of the articular involvement of posterior malleolar fractures on lateral radiographs and 2D CT images is shown. (a) Measurement on plain radiographs is shown practically. If we assume the horizontal distance between the front and back points of residual articular surface at the distal end of tibia is i and the horizontal distance between the posterior malleolar fracture line and the back point of articular surface is ii, then the percentage of involved articular surface is ii/i + ii. (b) When using axial CT linear measurement method, if we assume the distance between the front and back points of the lateral margin of residual articular surface at the level of tibial plafond is i and the distance between the posterior malleolar fracture line and the posterior point of lateral margin of the fragment is ii, then the percentage of involved articular surface is ii/i + ii. (c) When using sagittal CT linear measurement method, the process is similar to that on lateral radiographs. The percentage of involved articular surface is ii/i + ii. (d) When using axial CT plane measurement method, the boundary of the medial malleolus should be revealed at the same level. The boundary of the posterior malleolar fragment plane (ii) and residual plane (avoid medial malleolus area) (i) was delineated manually. Each area was calculated automatically by the software. Then the ratio of the involved fragment area is ii/i + ii.

In phase one, observers were asked to measure lateral radiographs of all the cases (Figure 2(a)) [14]. In phase two, axial CT images were used. The measurement was performed at the level of the tibial plafond (Figure 2(b)). In phase three, sagittal reconstruction images were used. The measurement was performed on the section of the fibular notch (Figure 2(c)). In the first three phases, the size of posterior malleolar fragment was measured as the percentage of the involved distal tibial articular surface (Figure 1(a)) [14, 15, 27].

In phase four, axial CT images were used. The measurement was performed at the level of the tibial plafond. The medial malleolus area should be revealed at the same level. The posterior malleolar fragment area and the remaining cross-sectional area (avoid medial malleolus area) of the tibia were delineated and measured. The ratio was then calculated as the fragment area to the total cross-sectional area of the tibial plafond (Figure 2(d)).

**Figure 3:**
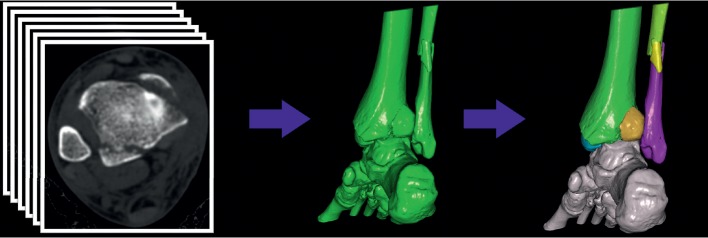
Three-dimensional processing of thin-section axial CT images is shown. Three-dimensional images (surface shaded display) were generated, and all component bones were distinguished by different colors.

**Figure 4:**
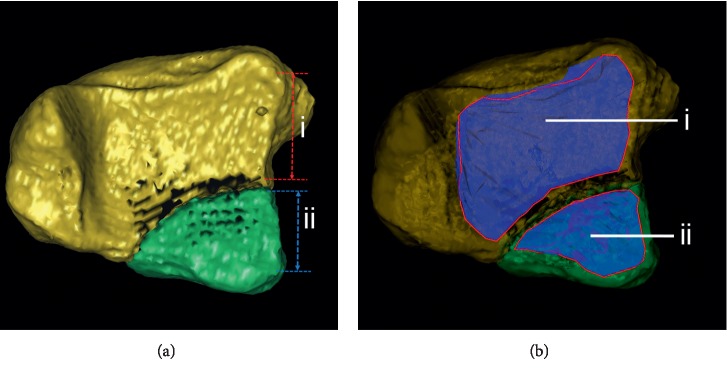
Measurement of the articular involvement of posterior malleolar fractures on 3D CT image is shown. (a) When using 3D CT linear measurement method, on the lateral border of the tibial plafond, if we assume the distance between front and back points of the residual articular surface is i and the distance between posterior malleolar fracture line and back point is ii, then the percentage of involved articular surface is ii/i + ii. (b) When using 3D CT surface measurement method, the surface boundary of the posterior malleolar fragment (ii) and residual articular surface (i) was delineated manually. Each surface area was calculated automatically by the software. Then, the ratio of the involved fragment area is ii/i + ii.

In phase five and six, 3D CT images were used. To perform 3D measurement, 3D images were firstly generated by surface shaded display (SSD) algorithm with a reconstruction interval of 0.625 mm. Secondly, all bones and fracture fragments were distinguished using the built-in interactive intelligent segmentation module (Figure 3). We hid the talus and turned over the distal tibial articular surface. In phase five, the size of posterior malleolar fragment was measured as the percentage of the involved distal tibial articular surface (Figure 4(a)). In phase six, the surface boundary of the posterior malleolar fragments and residual articular surface was delineated manually. Each surface area (along the curved plane of the distal tibia summing the surface areas from the separate transverse images) was calculated automatically by the software. The ratio of the posterior malleolar fragment area to the total area of the tibial plafond was calculated (Figure 4(b)).

### 2.3. Statistical Analysis

Statistical analysis was performed using SPSS 18.0 (SPSS Inc, Chicago, IL, USA) and MedCalc 15.10.0 (MedCalc Software bvba, Ostend, Belgium). The difference between the six measurement methods was analyzed using Bonferroni-corrected paired *t*-tests after one-way ANOVA. Only those *P* < 0.05/15=0.0033 were considered statistically significant. The agreement between the six measurement methods was analyzed using Bland–Altman analysis. The intraclass correlation coefficient (ICC, two-way mixed, single consistency) was used to assess intraobserver reproducibility and interobserver reliability [28]. The standard error of measurement (SEM) of the repeated measurements was calculated to determine the size of the measurement error. The SEM could be estimated as the square root of the mean square error term from the two-way random-effect ANOVA [29].

## 3. Results

### 3.1. Articular Involvement Determined Using Six Measurement Methods

One-way ANOVA showed a significant difference between ratios determined using six measurement methods (*F* = 31.379, *P* < 0.001). Significant differences were observed between values of any two of the six measurement methods (*P* < 0.0033), except between plane radiograph linear and axial CT linear (*t* = 1.574, *P*=0.118), between plane radiograph linear and sagittal CT linear (*t* = 1.471, *P*=0.144), between plane radiograph linear and 3D CT linear (*t* = 2.339, *P*=0.021), between axial CT linear and sagittal CT linear (*t* = 0.468, *P*=0.641), between axial CT linear and 3D CT linear (*t* = 2.567, *P*=0.012), and between sagittal CT linear and 3D CT linear (*t* = 1.885, *P*=0.062). The mean difference between plain radiograph linear and 3D CT surface was 8.58% (*t* = 10.564, *P* < 0.0033). The mean ratio determined using 3D CT surface was the smallest (16.0 ± 8.4%) and that of 3D CT linear was the largest (26.7 ± 8.3%) (Table 2). Compared to 3D CT linear, the articular involvement measured using plain radiographs and CT linear decreased by 8% and about 3%, respectively. Compared to 3D CT surface, the articular involvement measured using axial CT plane increased by 17.3% (Table 2).

**Figure 5:**
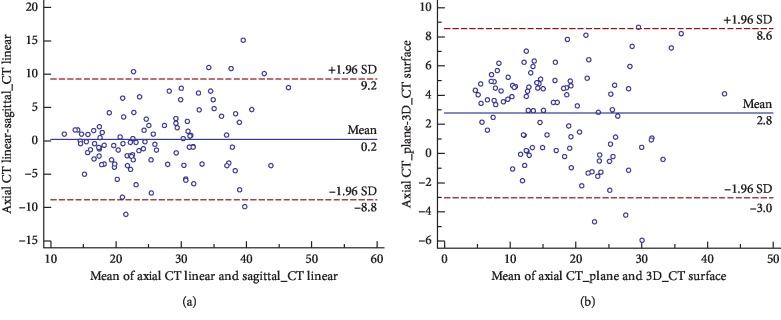
(a) Bland–Altman plot of axial CT linear and sagittal CT linear shows the 95% limits of agreement. (b) Bland–Altman plot of axial CT plane and 3D CT surface shows the 95% limits of agreement.

The Bland–Altman plots demonstrated poor agreement between values of any two of the six measurement methods. Within the range of 95% limits of agreement (95% LoA), maximum difference of more than 10% could be observed on most plots except for axial CT linear-sagittal CT linear, axial CT linear-3D CT linear, and axial CT plane-3D CT surface plot. The axial CT linear-sagittal CT linear plot showed the lowest mean difference: 0.21% (95% LoA −8.82 to 9.24%; 95% confidence interval for the bias −0.68 to 1.10%) (Table 3) (Figure 5(a)). The axial CT plane-3D CT surface plot showed the smallest difference interval: 95% LoA was 3.02 to 8.55% (Figure 5(b)).

### 3.2. Reliability and Reproducibility for Measurements

For fracture size measurement, the lowest intraobserver reproducibility was 0.46 (moderate) for resident surgeon using plain radiographs, and it improved to substantial (ICC = 0.77) for attending surgeon and to almost perfect (ICC = 0.82) for chief surgeon using plain radiographs. The intraobserver reproducibility for three surgeons using axial CT linear, sagittal CT linear, axial CT plane, 3D CT linear, and 3D CT surface was almost perfect (ICC, 0.82–0.96). For chief surgeon, the intraobserver reproducibility was almost perfect. And the ICC increased from 0.82 using plain radiographs to 0.94 using 3D CT surface (Table 4).

The lowest interobserver reliability was 0.41 (moderate) between chief and attending surgeon using plain radiographs, and it improved to substantial (ICC, 0.68–0.79) with the use of axial CT linear and to almost perfect (ICC, 0.81–0.95) with the use of 3D CT linear and 3D CT surface (Table 4).

The standard error of measurement showed almost the same results as ICC values. The lowest intraobserver reliability was 7.49 for resident surgeon using plain radiographs, while the highest was 1.91 for attending surgeon using 3D CT surface. The lowest interobserver reliability was 7.51 between chief and resident surgeon using plain radiographs, while the highest was 1.81 between chief and attending surgeon using 3D CT surface (Table 5).

## 4. Discussion

Up to now, controversy still remains on the operative indication for posterior malleolar fractures [6], and various indications were proposed which were based on a specific percentage measured by plain radiographs [7–14]. However, the operative indications will have no reference value if the measurement method itself is not reliable or reproducible. Therefore, the comparison of the results, reliability, and reproducibility of various methods for measuring posterior malleolar articular involvement is of great significance. In this study, significant differences were observed between values of any two of the six measurement methods, except between any two of the plane radiograph linear, axial CT linear, sagittal CT linear, and 3D CT linear. Poor agreement between values of any two of the six methods was observed. Three-dimensional CT showed the highest intraobserver reproducibility and interobserver reliability among three imaging modalities, while plain radiography revealed the lowest.

For diagnosing posterior malleolar fractures in our study, surgeons could not identify fracture line in 39 cases (18.6%) using plain radiographs. The fracture line of posterior malleolus was hard to identify as the overlap of the distal tibia and fibula. Therefore, for patients with confirmed or suspected ankle fracture, the potential risk of missing diagnosis of posterior malleolar fracture makes further CT examination necessary [15]. Some researchers recommended to diagnose and measure posterior malleolar fractures with 50 degrees external rotation lateral view [30]. Haraguchi type I fractures are oblique, but Haraguchi type II fractures are almost parallel to the coronal plane [17]. Due to the diversity of fracture lines of posterior malleolus, we doubt if one lateral view can satisfy all situations. In addition, patients may not cooperate because of pain and swelling of the ankle in the acute injury.

Plain radiographs are neither adequate in the diagnosis of posterior malleolar fractures nor reliable in assessing the posterior articular involvement. Ferries et al. [16] found a big difference between plain radiography and axial CT in the measurement of the articular involvement of posterior malleolar fractures. About 54% of the plain radiographic readings revealed >25% error. Meijer et al. [18] claimed a mean difference of 10.9% between plain radiographs and 3D CT images. In our study, similar findings were achieved. The mean difference was 5.82% between plain radiograph linear and axial CT plane and 8.58% between plain radiograph linear and 3D CT surface. The involvement of the fragment depends largely on which measurement method is used. Therefore, the existing operative indications determined based on plain radiography are not suitable for other measurement methods.

Some studies suggested that morphology of the posterior malleolar fragment might be more important than size for clinical decision making [21, 31]. Haraguchi type II fracture was also considered to be posterior pilon fracture, which has more complex mechanism and more special morphological characteristics [32]. Therefore, patients with Haraguchi type II fractures were not enrolled in this study. However, the morphology of posterior malleolus is hard to be determined using plain radiographs. We can only account on unapparent indications, such as a double-sign, to indicate the presence of Haraguchi type II fracture [31]. Thus, CT is useful not only in judging whether there is a fracture but also in identifying fracture morphology.

In our study, the level of intraobserver reproducibility of resident surgeon and all interobserver reliability for size measurement when based on plain radiographs was not clinically accepted. The previous study also concluded the impossibility to assess accurately the size of the posterior malleolar fragment on plain radiographs [17]. Meijer et al. [18] reported an interobserver agreement (ICC) of 0.61 on plain radiographs, which was similar to our results. A possible explanation of substantial bias is the obliqueness of the fracture line to the X-ray beam [10, 33]. Previous operative indications such as 25% and 33% were all set based on plain radiographs [7–9, 12–14]. The clinical significance of these indications is doubtful because the measurement method itself is unreliable and unrepeatable. Although the intraobserver reproducibility was almost perfect using 2D CT images (axial CT linear, sagittal CT linear, and axial CT plane), the interobserver reliability was substantial. Two main problems in measuring with CT images are important. Firstly, different from plain radiographs and 3D CT images, the measurement level of 2D CT would be determined by each observer. This might reduce the reliability and reproducibility. Secondly, when measuring the total cross-sectional area at the level of the tibial plafond, some previous studies overestimated the denominator (the total area of the tibial plafond) as it contains the area of the medial malleolus [16, 20]. When 3D CT was used, the intra- and interobserver agreement was stable and acceptable. Distal tibial articular surface cannot be observed by traditional imaging technique under direct vision because of obstruction by talus. The SSD technique, which had been proved to be superior in bony surface reconstructions, was used to distinguish bony structures in our study [24, 34]. The tibial plafond was revealed and measured under direct vision after hiding the talus. Therefore, the operative indications need to be reevaluated with the use of a more reliable and reproducible measurement method such as 3D CT linear or 3D CT surface methods.

The above measurement methods can be summarized into two categories. One category is to measure the ratio of the lateral margin of the posterior malleolar fragment to the total fibular notch (plain radiograph, axial CT linear, sagittal CT linear, and 3D CT linear), which was in line with the traditional method [14]. The other is to measure the ratio of the area of the fragment to the total tibial plafond (axial CT plane and 3D CT surface). The 3D CT linear measurement method from the first category and the 3D CT surface measurement method from the second category were proved to be reliable and reproducible. However, linear measurement could only detect the injury degree of the fibular notch and could not show the true injury area of the joint surface.

Interestingly, the results revealed that the level of experience was not so important to intraobserver reproducibility when using 3D CT images. One may guess that more experience would result in better consistency in measuring fracture involvement. When based on plain radiographs, experience is exactly crucial. The intraobserver reproducibility of resident surgeon cannot be clinically accepted. However, using 3D CT images was not the case. The level of intra- and interobserver agreement was similar among three surgeons. The level of intraobserver reproducibility of resident surgeon was significantly enhanced.

The current study had some limitations. Firstly, we only included patients with striking posterior malleolar fracture line on lateral plain radiographs. Therefore, the ICC might be higher than those in published reports. Besides, a small part of the patients were excluded from the samples because of severe comminution or defect of posterior malleolar fragments on 3D CT images. Continuous further studies are required to solve these problems.

## 5. Conclusions

The existing operative indications which were determined based on plain radiography are neither reliable nor suitable for other measurement methods. Both 3D linear and 3D surface measurement method are reliable and reproducible in measuring posterior fragment involvement, and experience is not so crucial. We call on further multicenter clinical research on large sample to demonstrate the relationship between prognosis and posterior malleolar fracture involvement on the basis of 3D CT images, in order to redefine the operative indications for posterior malleolar fractures.

## Figures and Tables

**Table 1:** Demographic data of the patients (*n* = 106).

Characteristic	Value
Age, *y* (range)	47.3 (21 to 75)
Male, *n* (%)	59 (55.7)
Left, *n* (%)	45 (42.5)
AO/OTA classification, *n* (%)	
44-B	75 (70.8)
44-C	31 (29.2)
Lauge-Hansen classification, *n* (%)	
Supination-external rotation	
Type 3	31 (29.2)
Type 4	43 (40.6)
Pronation-external rotation type 4	27 (25.5)
Pronation-abduction type 3	5 (4.7)

**Table 2:** Fracture involvement determined using six measurement methods.

Measurement method	Fracture involvement in mean ± SD (%)	95% CI
Plane radiograph linear	24.6 ± 7.9	23.1–26.1
Axial CT linear	26.0 ± 8.9	24.3–27.7
Sagittal CT linear	25.8 ± 7.8	24.3–27.3
Axial CT plane	18.8 ± 7.9	17.3–20.3
3D CT linear	26.7 ± 8.3	25.1–28.3
3D CT surface	16.0 ± 8.4	14.4–17.6

**Table 3:** Agreement between values of any two of the six measurement methods.

	Axial CT linear	Sagittal CT linear	Axial CT plane	3D CT linear	3D CT surface
Plain radiograph linear	5.82, −10.75 to 22.38	−1.22, −17.89 to 15.46	−1.42, −19.69 to 16.84	−2.13, −20.52 to 16.25	8.58, −7.81 to 24.98
Axial CT linear		0.21, −8.82 to 9.24	7.24, 1.21 to 13.28	−0.71, −6.26 to 4.84	10.00, 3.88 to 16.13
Sagittal CT linear			7.03, −1.17 to 15.24	−0.92, −10.72 to 8.89	9.80, 2.87 to 16.73
Axial CT plane				−7.95, −15.83 to −0.07	2.77, 3.02 to 8.55
3D CT linear					10.71, 2.72 to 18.71

Data are presented as mean difference (%), Bland–Altman 95% limits of agreement (mean difference ± 1.96 SD) (%).

**Table 4:** Inter- and intraobserver agreement for fracture involvement determined using six measurement methods.

Examiner	Plain radiographs	Axial CT linear	Sagittal CT linear	Axial CT plane	3D CT linear	3D CT surface
Chief-chief	0.82 (0.74–0.87)	0.89 (0.85–0.93)	0.88 (0.83–0.92)	0.92 (0.88–0.94)	0.91 (0.88–0.94)	0.94 (0.92–0.96)
Attending-attending	0.77 (0.68–0.84)	0.92 (0.88–0.94)	0.85 (0.78–0.89)	0.96 (0.94–0.97)	0.91 (0.87–0.94)	0.95 (0.93–0.97)
Resident-resident	0.46 (0.30–0.60)	0.90 (0.86–0.93)	0.82 (0.75–0.88)	0.94 (0.91–0.96)	0.88 (0.82–0.91)	0.91 (0.88–0.94)
Chief-attending	0.41 (0.24–0.55)	0.79 (0.70–0.85)	0.73 (0.63–0.81)	0.66 (0.53–0.75)	0.84 (0.78–0.89)	0.95 (0.93–0.97)
Chief-resident	0.43 (0.26–0.57)	0.68 (0.56–0.77)	0.52 (0.37–0.65)	0.70 (0.59–0.78)	0.81 (0.74–0.87)	0.95 (0.93–0.97)
Attending-resident	0.67 (0.55–0.76)	0.70 (0.59–0.79)	0.59 (0.45–0.70)	0.60 (0.46–0.71)	0.83 (0.76–0.88)	0.90 (0.86–0.93)

Values are presented as intraclass correlation coefficient values with 95% confidence interval; agreement was divided into 6 levels, including almost perfect (0.81 to 1.00), substantial (0.61–0.80), moderate (0.41 to 0.60), fair (0.21 to 0.40), slight (0.00 to 0.20), and poor (below 0.00).

**Table 5:** Inter- and intraobserver reliability for fracture involvement determined using six measurement methods.

Examiner	Plain radiographs	Axial CT linear	Sagittal CT linear	Axial CT plane	3D CT linear	3D CT surface
Chief-chief	4.66	3.01	3.25	2.37	2.59	2.01
Attending-attending	4.79	3.18	3.62	2.11	2.92	1.91
Resident-resident	7.49	3.13	3.99	2.25	3.11	2.68
Chief-attending	7.49	4.60	4.60	5.28	3.55	1.81
Chief-resident	7.51	5.35	6.28	4.80	3.69	1.98
Attending-resident	5.46	5.59	5.90	5.88	3.75	2.79

Values are presented as standard error of measurement; the larger the standard error of measurement, the lower the reliability.

## Data Availability

The data used to support the findings of this study are available from the corresponding author upon request.

## Supplementary Materials

Supplementary Materials Figure S1: Bland–Altman plots and detailed comparison data of any two of the measurement methods (associated with the data of Table 3).
