# Supplementary figures and images for: Quantitative Evaluation of Articular Involvement of Posterior Malleolus Associated with Operative Indication: A Comparative Study of Six Methods Based on Radiography and CT
Source: Biomed Res Int. 2020 Jan 2;2020:6745626. doi: 10.1155/2020/6745626 (PMC6970486; doi:10.1155/2020/6745626)

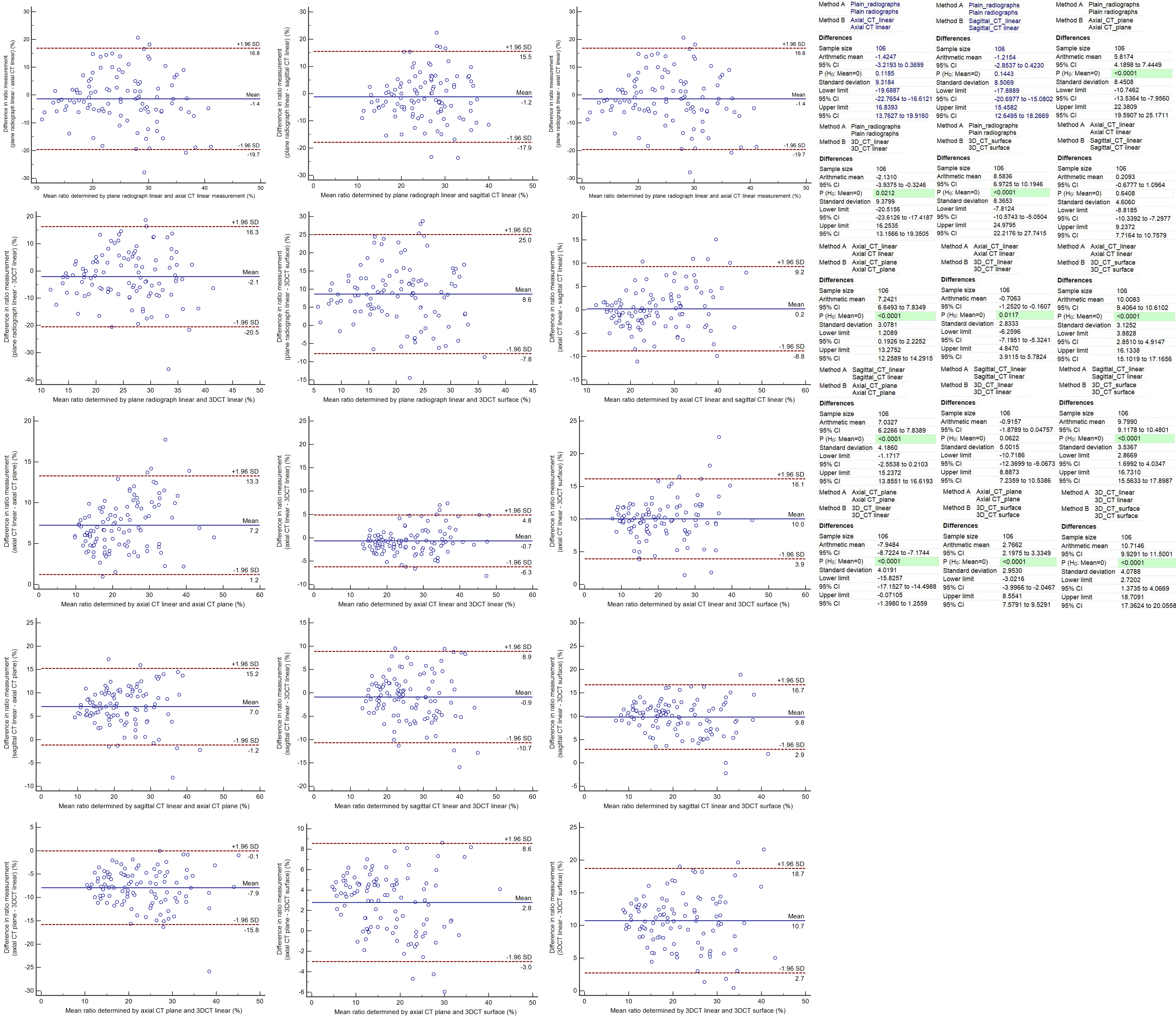

Supplement: Supplementary Materials — Figure S1: Bland–Altman plots and detailed comparison data of any two of the measurement methods (associated with the data of Table 3). [file 6745626.f1.tif]
